# The Characterization of *chIFITMs* in Avian Coronavirus Infection In Vivo, Ex Vivo and In Vitro

**DOI:** 10.3390/genes11080918

**Published:** 2020-08-10

**Authors:** Angela Steyn, Sarah Keep, Erica Bickerton, Mark Fife

**Affiliations:** 1 The Pirbright Institute, Pirbright, Woking GU24 0NF, UK; sarah.keep@pirbright.ac.uk (S.K.); erica.bickerton@pirbright.ac.uk (E.B); mfife@aviagen.com (M.F.); 2 AVIAGEN UK, Ltd. Newbridge, Midlothian EH28 8SZ, Scotland, UK

**Keywords:** coronavirus, avian infectious bronchitis virus (IBV), interferon-inducible transmembrane (IFITM) proteins

## Abstract

The coronaviruses are a large family of enveloped RNA viruses that commonly cause gastrointestinal or respiratory illnesses in the infected host. Avian coronavirus infectious bronchitis virus (IBV) is a highly contagious respiratory pathogen of chickens that can affect the kidneys and reproductive systems resulting in bird mortality and decreased reproductivity. The interferon-inducible transmembrane (IFITM) proteins are activated in response to viral infections and represent a class of cellular restriction factors that restrict the replication of many viral pathogens. Here, we characterize the relative mRNA expression of the chicken *IFITM* genes in response to IBV infection, in vivo, ex vivo and in vitro using the pathogenic M41-CK strain, the nephropathogenic QX strain and the nonpathogenic Beaudette strain. In vivo we demonstrate a significant upregulation of *chIFITM1*, *2*, *3* and *5* in M41-CK- and QX-infected trachea two days post-infection. In vitro infection with Beaudette, M41-CK and QX results in a significant upregulation of *chIFITM1*, *2* and *3* at 24 h post-infection. We confirmed a differential innate response following infection with distinct IBV strains and believe that our data provide new insights into the possible role of *chIFITMs* in early IBV infection.

## 1. Introduction

Coronaviruses encompass a large family of positive-stranded RNA viruses that cause a range of diseases in humans and animals [1]. The *Coronaviridae* family consists of two subfamilies, the *Letovirinae* and *Orthocoronavirinae*, the latter of which is divided into four genera named, *α-*, *β-*, *Delta-* and *Gammacoronavirus*. The avian coronavirus, infectious bronchitis virus (IBV) is the prototype *Gammacoronavirus*. Notable members of the *Betacoronavirus* genus include the human coronaviruses, murine hepatitis virus (MHV), Middle Eastern respiratory syndrome coronavirus (MERS-CoV) [1,2,3,4], severe acute respiratory syndrome coronavirus (SARS-CoV) [2] and the virus responsible for COVID-19, SARS-CoV-2 [3].

Infectious bronchitis virus infects domestic fowl (*Gallus gallus*) and is the causative agent of a highly contagious respiratory disease, Infectious Bronchitis (IB). IBV replicates primarily in the tracheal epithelial cells of the respiratory tract, resulting in a decrease in tracheal ciliary activity and common-cold-like symptoms including snicking, tracheal rales and nasal discharge [5]. Secondary replication of IBV can occur in many non-respiratory tissues including the kidney, testes, oviduct and gastrointestinal tract [6,7]. The clinical symptoms presented by infected birds are dependent on several factors including the strain of IBV, of which there are many genetic variants and serotypes. The majority of IBV strains, including the Massachusetts (Mass) serotype M41 produce prominent respiratory disease [8]. In contrast, QX, an IBV strain first isolated in China from chickens with proventriculitis [9], is considered nephropathogenic and in addition to tracheal lesions, infection also induces prominent kidney lesions. QX infection results in a higher mortality rate [8] than a classical respiratory infection. The common features of all IBV infections include lethargy, weight loss and reduced egg production and quality [6]. IBV infection is therefore of major economic importance to the poultry industry worldwide and despite intensive control measures, remains prevalent [10].

The IBV genome contains a positive-sense single-stranded RNA molecule of approximately 27.6 kb in length [11,12] which is polyadenylated at the 3′ end and contains a methylated cap at the 5′ end. The genome encodes 15 nonstructural proteins (nsp) that are expressed as part of two large polyproteins, pp1a and pp1ab, the latter is generated via a ribosomal frameshift [13,14]. The genome also encodes four major structural proteins; the nucleocapsid (N) protein, the membrane glycoprotein (M), a small envelope protein (E) and the spike glycoprotein (S), as well as four confirmed accessory proteins, 3a, 3b, 5a and 5b [15]. These proteins play different roles in the replication cycle with S notably mediating virus attachment and entry [16], thus being largely responsible for cell and host tropism [17,18,19]. The binding of a virus to the host cell is the first step in determining tissue and host specificity and ultimately pathogenicity [19,20]. Some viruses bind to abundant and universal host factors, resulting in the infection of various host species while other viruses, such as the coronaviruses, have a limited range of susceptible hosts [19,21]. Infectious bronchitis virus has a narrow host tropism and although this host tropism is restricted to chickens, the cell and tissue tropism in chickens differ substantially between different IBV strains [19,22].

Current IBV isolates present highly diverse antigenicity and emergent strains, that differ in antigenic properties, tissue tropism and pathogenicity, and are continuously being reported across the world [23,24]. IBV is currently controlled by the use of both live-attenuated and inactivated boost vaccine strains of varying serotypes such as Massachusetts, Connecticut and Arkansas. Chickens receive combinations of vaccine viruses in an effort to induce protective responses against multiple field strains; however, the cross-protection induced is limited and often short-lived [25,26,27,28]. In addition, the constant emergence of new variant IBV strains leads to outbreaks of the virus in vaccinated flocks resulting in significant economic losses to the poultry industry [7,29,30]. Therefore, the use of innate immune mediators to boost the innate immune system may be an alternative approach to control IBV infection [31].

In response to viral infection, nearly all vertebrate cells activate the innate immune response as a first line of defense [32,33]. The group of cytokines produced, type I interferons (IFNs), function to induce the expression of a cascade of interferon-stimulated genes (ISGs) resulting in an antiviral state [34]. These ISGs encode antiviral proteins that inhibit several stages of the viral lifecycle including viral entry, translation, replication, assembly and egress [35,36]. The interferon-inducible transmembrane proteins (IFITMs) are widely expressed IFN-inducible proteins that restrict the entry and therefore infection, by multiple pathogenic viruses including, influenza A virus (IAV), West Nile virus (WNV), dengue virus (DENV), severe acute respiratory syndrome coronavirus (SARS-CoV), vesicular stomatitis virus (VSV) and hepatitis C virus (HCV), among others [34,35,37,38,39,40,41,42,43,44]. Many studies have aimed to understand the mechanisms behind the antiviral activity of the IFITM proteins [39,45,46,47,48]. Early work showed that IFITMs are located on the endosomal membranes and block viral particles that have the receptors of restricted viruses [40]. Later work suggested that IFITMs restrict viral entry by changing the properties of cellular membranes [49] resulting in IFITM-sensitive viruses being blocked at the cell surface and becoming trapped in the endosomal pathway, ultimately preventing viral fusion [40,45].

The IFITM protein family is encoded by five genes in humans, including the immune-related *IFITM1*, *IFITM2* and *IFITM3*, as well as *IFITM5* and *IFITM10*, which have no characterized roles in immunity [36]. IFITMs are present in a wide range of species: from amphibians, fish and birds to mammals. The chicken *IFITM* (*chIFITM*) genes are clustered on chromosome 5, and to date, four genes have been annotated, namely *chIFITM1*, *chIFITM3*, *chIFITM5* and *chIFITM10* [50]. Although not yet annotated in the *Gallus gallus* reference sequence, studies show the existence of *chIFITM2* and suggest a hypothetical genetic structure of the locus based on the human syntenic genome region [51]. The mammalian IFITM proteins are relatively small (about 130 amino acids) [33] and share a topology defined by a conserved CD225 domain, consisting of two intramembrane (IM) regions and a conserved intracellular loop (CIL) [52]. The *chIFITMs* show significant sequence divergence from mammals, and as such their topology is less well defined. The unique ability of IFITMs to inhibit viral entry into the host cell highlights their importance in innate immunity; by preventing cell entry, they subsequently prevent viral replication and disease [53].

Despite the progress in understanding the IFITM-mediated antiviral capacity, host *chIFITM* expression and viral restriction profiles in response to IBV infection have yet to be elucidated.

In this study, we observed significant changes in the transcriptional levels of *chIFITMs* in vivo, in vitro and ex vivo, after infection with three strains of IBV that vary in pathogenicity and tissue tropism, suggesting a potential role in the early defense against IBV infection.

## 2. Materials and Methods

### 2.1. Ethics Statement

All animal experiments have been published previously [54,55] and were carried out following the UK Home Office guidelines and the UK Animals (Scientific Procedures) Act 1986. All experiments were performed at The Pirbright Institute animal facilities, license number X24684464.

### 2.2. Viruses and Cells

The IBV strains used were (i) the pathogenic M41-CK strain (GenBank accession number MK728875.1), derived from the pathogenic M41 strain after multiple passages in primary chick kidney (CK) cells and displays classical respiratory disease [56]; (ii) the nonpathogenic recombinant laboratory strain Beau-R (GenBank accession number AJ311317), a molecular clone of CK cell-culture-adapted Beaudette strain [5]; (iii) the nephropathogenic QX strain (GenBank accession number KY933090). We also used a QX-like strain, D388 [57], for in vitro studies as QX exhibits limited tropism and does not infect DF-1 cells. The M41-CK isolate is able to produce infectious virions in CK cells but not in DF-1 cells, while Beau-R exhibits extended cell tropism and is able to produce infectious virions in both cell types [5,18,58]. The QX strain is not able to produce infectious virions in either chicken fibroblast cell line, DF-1 or CK cells [59]. All viruses were propagated in embryonated hen’s eggs and quantified via titration in triplicate in either CK cells [60,61,62] or ex vivo tracheal organ cultures (TOCs) as described by [20]. Titers are displayed as the number of plaque-forming units (PFU) per mL or as a 50% (median) ciliostatic dose (CD_50_) per mL.

Primary CK cells were produced by the Central Services Unit (CSU) at The Pirbright Institute (TPI). CK cells were prepared from kidneys extracted from three-week-old specific pathogen-free (SPF) Rhode Island Red (RIR) chickens following a method previously described by [63]. DF-1 (immortalized chicken embryo fibroblast cell line) cells were obtained from CSU at TPI. All cell cultures were maintained at 37 °C and 5% CO_2_. Chickens were hatched and reared at TPI. Tracheal organ cultures were prepared from 19-day-old RIR SPF embryos as described by [64]. Cultures were maintained at 37 °C, rotating at 7–8 revolutions per hour.

### 2.3. Virus Infection In Vivo

The tissues used for the in vivo analysis of this study were harvested as part of two previously described studies thereby incorporating the principles of the 3Rs: Replacement, Reduction and Refinement.

Briefly, in the first study, groups of 24 8-day old SPF RIR chickens were inoculated with either phosphate-buffered saline (PBS) (mock) or 10^5^ plaque-forming units (PFU) of either nonpathogenic recombinant (rIBV) Beau-R or pathogenic IBV, M41-CK. Clinical signs, including snicking and rales, were assessed from 1 to 7 days post-infection (dpi). Snicks were individually counted by two persons over a two-minute period and birds were checked individually for the presence of tracheal rales. On 1, 4, 6 and 7 dpi, 6 randomly selected birds were euthanized by cervical dislocation and trachea tissue harvested. Ciliary activity in the trachea was assessed on 4 and 6 dpi [55]. Tracheal ciliary activity is used as the gold standard to determine the pathogenicity of an IBV strain [65], a ciliary activity score of less than 50% is deemed pathogenic. The clinical signs and ciliary activity have previously been reported [55].

In the second study, groups of 30 8-day old SPF RIR chicks were vaccinated with PBS via the intraocular and intranasal route [54]. Three weeks postvaccination chickens were challenged, via the same route of administration with either PBS, 10^5^ PFU M41-CK, or 10^2.73^ CD_50_ nephropathogenic strain QX. Two, 4- and 14-days post-challenge, 5 birds were euthanized by cervical dislocation and a panel of tissues harvested, of which we used trachea and kidney for the present study. Kidney tissue was not harvested at 14 dpi. Clinical signs were assessed from days 2 to 8 post-challenge, and then on day 14. Ciliary activity was measured at day 4 post-infection. The clinical signs and ciliary activity have previously been reported [54]. A schematic overview of both study designs can be found in the supplementary data (Supplementary Figure S1). All tissue samples were stored at −80 °C in RNAlater solution.

### 2.4. Virus Infection In Vitro

Confluent CK cells seeded in 12-well plates were infected with 1 × 10^6^ PFU/mL of Beau-R, M41-CK or D388 (MOI = 1), or mock infected with sterile PBS and incubated at 37 °C and 5% CO_2_ for 1 h before washing with PBS and replacing with 1 mL maintenance media containing 20 mM BES (*N*,*N*-Bis(2-hydroxyethyl)-2aminoethanesulphonic acid) (Sigma, St Louis, MO, USA, purity ≥99%), 1X Eagle’s Minimum Essential Medium (EMEM), 10% TPB (Tryptose Phosphate Broth, 0.2% Bovine Serum Albumin (BSA); 0.21% sodium bicarbonate, 2 mM L-glutamine, 50 U/mL nystatin, 10 U/mL penicillin and 10 µg/mL streptomycin. The BES is filtered sterilized before being incorporated into the medium [66]. 

DF-1 cells were infected with Beau-R (MOI = 1) or mock infected with sterile PBS and incubated at 37 °C and 5% CO_2_ for 1 h before washing with PBS and replacing with 1 mL maintenance media. Total cellular RNA was extracted from CKC and DF-1 cell lysates harvested at 12, 24 and 36 and 6, 12, 24, 36 and 48 hours post infection (hpi) respectively. Viral supernatants were obtained and viral titers were determined by standard plaque assay in CK cells. 

### 2.5. Virus Infection in Ex Vivo Tracheal Organ Cultures (TOCs)

TOCs were prepared from 2- to 3-week-old RIR SPF chickens. TOCs (one tracheal ring per tube) were infected 4 days after preparation to allow the early inflammatory responses of the tissue to subside [24,67]. TOCs were infected for one hour with either 1 × 10^5^ PFU/mL Beau-R or M41-CK, or with 10^2.73^ CD_50_ QX and 10^2.73^ M41-CK, or mock infected with TOC medium without virus. TOC rings and supernatant were harvested at 2, 4 and 8 hpi and stored at −80 °C. This experiment was performed in duplicate. The viral titer from harvested inoculum from Beau-R- and M41-CK-infected TOCs was determined by plaque assay titration on primary CK cells. The harvested inoculum from IBV M41-CK and QX strains were titrated in ex vivo TOCS [20] due to the restricted tropism of the QX strain.

### 2.6. Total RNA Extraction and Two-Step Reverse Transcription

Total RNA from tissue samples and cell pellets were extracted using the RNeasy Kit (Qiagen, Manchester, UK) following the manufacturer’s instructions (for RNA extraction from animal tissues and animal cells) and included an on-column DNAse treatment step using the Rnase-free Dnase kit (Qiagen, Manchester, UK). RNA concentration was quantified by Nanodrop (Thermo Scientific). One µg of RNA was reverse transcribed into cDNA using SuperScript III First-Strand cDNA synthesis with random primers following the manufacturer’s instructions. The cDNA concentration of each sample was normalized to 100 ng/µL in nuclease-free water for all quantitative reverse transcription PCR reactions. The presence of IBV was tested in all RNA samples using primers BG-56 (5′-CAACAGCGCCCAAAGAAG-3′) and 93/100 (5′-GCTCTAACTCTATACTAGCCT-3′) that target the 3′ untranslated region (UTR) of each IBV strain. PCR amplicons were run on a 2% gel and run for 1 h at 120 V. These oligonucleotides can discriminate between the 3′ UTRs from Beau-R and M41-CK derived sequences. The 3′ UTR of QX and Beau-R results in a 667 bp product but a 483 bp PCR from M41-CK due to a 184 nt deletion in the M41 3′ UTR [68].

### 2.7. Quantification of Virus Subgenomic RNA (sgRNA) in Infected Tissues, Cells and TOCs

Viral RNA load was quantified by qRT-PCR using Taqman Fast Universal PCR 2× Master Mix and including 125 nM final probe and 500 nM final primers. These primers and probe (taken from [69]) specifically target the IBV N message (sgRNA) of Beau-R, M41-CK and QX IBV strains (one bp mismatch in reverse primer for QX strain still successfully and specifically amplified the QX sgRNA). Leader Forward Primer: 5′-CTAGCCTTGCGCTAGATTTTTAACT-3′; N sgRNA Reverse Primer 5′-GAGAGGTACACGCGGGACAA-3′; IBV sgRNA probe 5′-FAM-ACAAAGCAGGACAAGCA-MGB-NFQ-3′. Absolute quantitation of IBV RNA copy numbers was performed using standard curves generated by a serial dilution of plasmids. The C_T_ results were used to calculate the log RNA copies (Log_10_) using the linear equation from the standard curve.

### 2.8. IFITM Gene Expression Analysis

Primer and probe assays to specifically target each of the *chIFITM* genes were designed by Primerdesign. Briefly, 1 µL of the *chIFITM* assay was added to 5 µL Taqman Fast Universal PCR 2× Master Mix, 2 µL ddH20 and 2 µL cDNA. The cycling conditions were 10 min at 95 °C, 35 cycles of 10 s at 95 °C, 10 s at 60 °C annealing temperature and 10 s at 75 °C. qRT-PCR data were normalized using two housekeeping genes, *RPLPO* and *RPL13* [70]. Data are presented as the fold change in relative mRNA gene expression of virus-infected versus mock-infected samples. Analysis was performed using the relative quantification of gene expression (∆∆C_T_, where C_T_ is the cycle threshold) using the QuantStudio 5 software. The target genes were compared to these two housekeeping genes individually and the ΔΔC_T_ averaged. The data were analyzed in GraphPad Prism using one-way ANOVA. ANOVA data were corrected for multiple comparisons using the Bonferroni adjustment method. Differences between groups at that time point were considered significant at *p* < 0.05.

## 3. Results

### 3.1. Clinical Observation

In the first study, mock-infected birds and those infected with rIBV Beau-R, a molecular clone of the pathogenic Beau-CK strain [5], displayed no IBV-related clinical signs and no reduction in ciliary activity while birds infected with M41-CK displayed clinical signs from day 2 post-inoculation and had a reduced ciliary activity (<2%), as previously described in [55].

In the second study, both QX- and M41-CK-infected birds exhibited IB-related symptoms, including snicking and rales, and reduction in ciliary activity as previously described [54]. The reduction in ciliary activity and the observed clinical signs further confirms the in vivo pathogenic phenotype of both these IBV strains [54].

### 3.2. Transcriptional Profile of chIFITMs In Vivo

To determine whether IBV infection induces *chIFITM* expression in vivo, tissues from chickens infected with three strains of IBV of different pathogenicities, were investigated; the apathogenic attenuated Beau-R strain, pathogenic laboratory M41-CK strain and the nephropathogenic QX strain were compared. The relative mRNA expression of *IFN-α*, *IFN-β*, *chIFITM1*, *2*, *3* and *5* as well as *Mx* was measured via qRT-PCR using cDNA originating from the RNA extracted from infected tissue. *Mx*, a gene that is highly upregulated in response to viral infection, was used as a positive control for all qPCR experiments.

**Figure 1:**
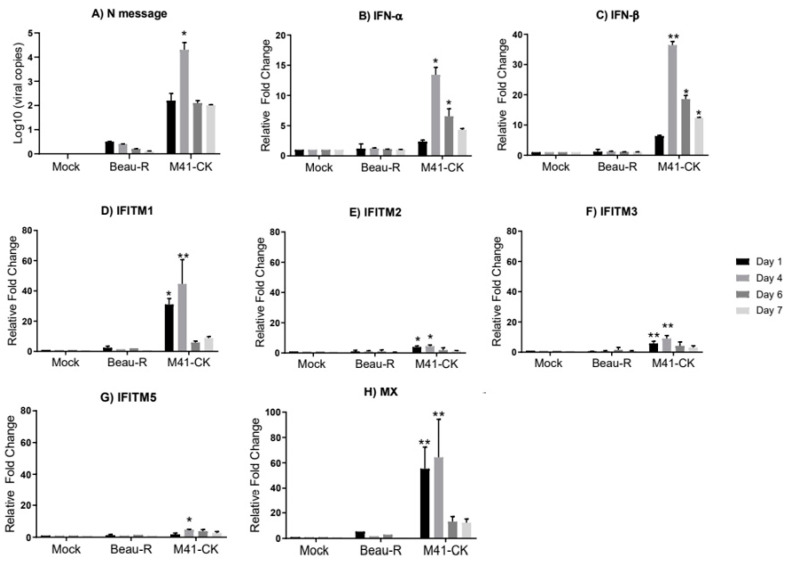
Expression of *IFNs* and *chIFITMs* are upregulated in the trachea of chickens in response to M41-CK infection but not to Beau-R infection. (**A**) Log_10_ of infectious bronchitis virus (IBV) RNA copies in tracheas from chickens infected with Beau-R and M41-CK. The IBV genome loads were determined using absolute quantification. Relative mRNA expression of (**B**) *IFN-α*, (**C**) *IFN-β*, (**D**–**G**) *chIFITM1*, *2*, *3* and *5* and (**H**) *Mx* in trachea from chickens infected with pathogenic M41-CK and nonpathogenic Beau-R strain of IBV were analyzed for gene expression at 1, 4, 6 and 7 days post infection (dpi). Relative mRNA expression was determined by real-time PCR normalized to two reference genes *RPLPO* and *RPL13*. All graph values are the mean of three biological replicates with error bars as standard deviation; * indicates *p* < 0.05, ** indicates *p* < 0.01 ANOVA data were corrected for multiple comparisons using the Bonferroni adjustment method (GraphPad).

We did not detect any viral replication of Beau-R in infected trachea; the Beau-R genome copy number in the infected trachea was comparable to levels observed in mock-infected samples (Figure 1A). The absence of replicating Beau-R in the trachea supports what has been seen in previous studies [20,69,71]. Infection with Beau-R did not elicit an upregulation of either *IFN-α*, *IFN-β* or *chIFITMs* mRNA in infected trachea (Figure 1B–G), strengthening the assumption that Beau-R fails to produce a productive infection in vivo [55]. In the trachea of chickens infected with M41-CK, the viral copy number was significantly higher when compared to mock-infected and Beau-R-infected samples, peaking at 4 dpi (9.8 × 10^4^ ± 1.5 × 10^5^ copies/µL; *p* = 0.017), steadily decreasing until 7 dpi (1.28 × 10^2^ ± 2.7 × 10^1^ copies/µL) (Figure 1A). In the M41-CK-infected trachea, both *IFN-α* and *IFN-β* are significantly upregulated, with relative mRNA expression peaking at 4 dpi for both (Figure 1B,C, respectively), consistent with IBV replication which also peaks at 4 dpi. *chIFITM1, 2* and *3* are significantly upregulated at 1 dpi and at 4 dpi, decreasing until levels are comparable to that observed in mock-infected samples at 7 dpi (Figure 1D–F). The upregulation of *chIFITM5* was only significantly upregulated in comparison to mock- and Beau-R-infected trachea at 4 dpi (Figure 1G). Due to the observation that Beau-R does not produce a productive infection in vivo, and therefore does not elicit an interferon or a *chIFITM* response, a nephropathogenic strain of IBV, QX, was included in the analysis. This enables a valuable comparison of *chIFITM* response in IBV strains of varying pathogenicities.

**Figure 2:**
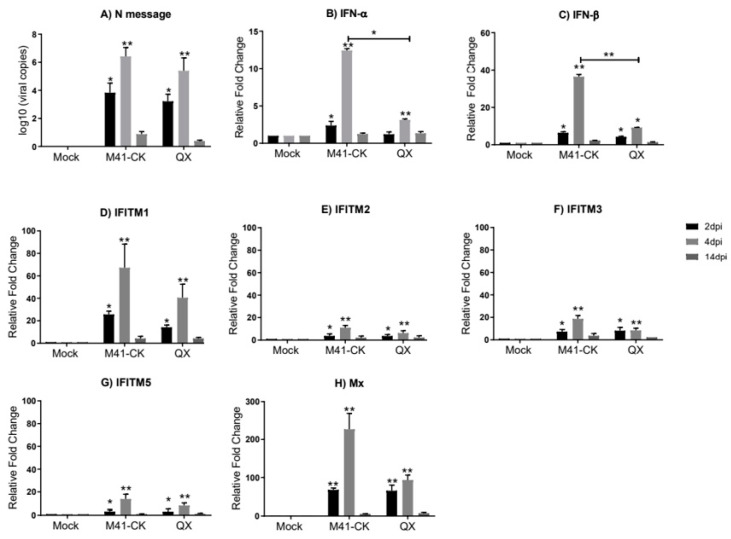
Relative expression of *IFNs* and *chIFITMs* in trachea samples from chickens infected with M41-CK and the nephropathogenic QX strain. (**A**) Log_10_ of IBV RNA copies in trachea from chickens infected with pathogenic M41-CK or nephropathogenic QX, or mock infected, measured at 2, 4 and 14 dpi. Relative expression of (**B**) *IFN-α*, (**C**) *IFN-β* and (**D**–**G**) *chIFITM1*, *2*, *3* and *5* and (**H**) *Mx* measured by qRT-PCR in tracheal samples collected at 2, 4 and 14 dpi from chickens experimentally challenged with M41-CK and QX IBV, or mock infected. Relative fold change was calculated using the ∆∆CT equation, relative to mock and normalized to two reference genes, *RPLPO* and *RPL13*. All graph values are the mean of three biological replicates with error bars as standard deviation. * indicates *p* < 0.05, ** indicates *p* < 0.01. ANOVA data were corrected for multiple comparisons using the Bonferroni adjustment method (GraphPad).

In the comparison of M41-CK and QX IBV strains, we observed active replication of the virus in the trachea of infected chickens (Figure 2A). IBV-derived RNA was detectable as early as 2 dpi. The viral load for M41-CK and QX peaked at 4 dpi (5.3 × 10^4^ ± 8.6 × 10^3^ and 4.3 × 10^3^ ± 6.2 × 10^3^), respectively, and by 14 dpi, only low levels of replicating virus were observed. The viral load in the trachea detected in this study is comparable to the relative viral load detected by Ellis et al. [54].

**Figure 3:**
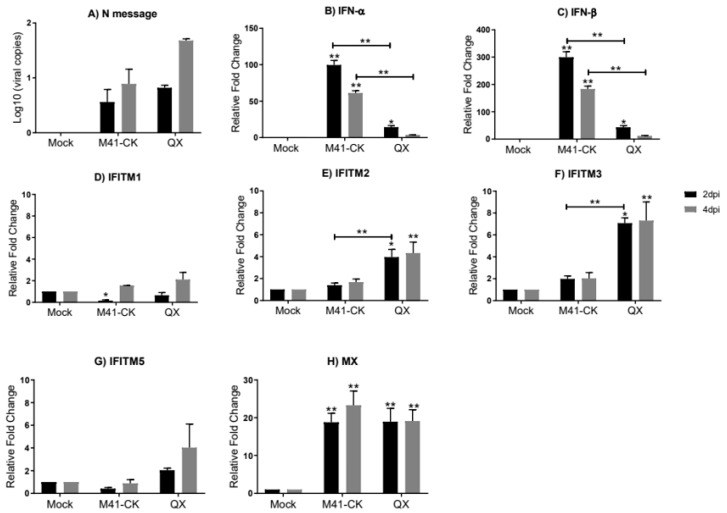
Relative expression of *chIFITMs* and *IFNs* in kidney samples from chickens infected with M41-CK and the nephropathogenic QX strain. (**A**) Log_10_ of IBV RNA copies in the kidney from chickens infected with pathogenic M41-CK or nephropathogenic QX, or mock infected, measured at 2 and 4 dpi. Relative expression of (**B**) *IFN-α*, (**C**) *IFN-β* and (**D**–**G**) *chIFITM1*, *2*, *3* and *5* and (**H**) *Mx* measured by qRT-PCR in kidney samples collected at 2 and 4 dpi from chickens experimentally challenged with M41-CK and QX IBV, or mock infected. Relative fold change was calculated using the ∆∆CT equation, relative to mock and normalized to two reference genes, *RPLPO* and *RPL13*. All graph values are the mean of three biological replicates with error bars as standard deviation. * indicates *p* < 0.05, ** indicates *p* < 0.01. ANOVA data were corrected for multiple comparisons using the Bonferroni adjustment method (GraphPad).

In trachea from M41-CK- and QX-infected chickens, although significantly upregulated as early as 2 dpi, the relative expression of IFN-*α*, *IFN-β* and *chIFITM1*, *2*, *3* and *5* peaks at 4 dpi (Figure 2B–G). This significant induction of immune genes is consistent with IBV replication which peaks at 4 dpi in infected trachea (Figure 2A). IFN-*α* and IFN-*β* both display significantly higher mRNA expression in M41-CK-infected trachea when compared to QX-infected trachea (*p*-value = 5.4 × 10^−2^ and 4.21 × 10^−3^, respectively). The *chIFITM* genes display a similar pattern of expression in M41-CK- and QX-infected trachea; the highest and most significant upregulation of *chIFITM* mRNA expression was observed at 4 dpi and had decreased by 14 dpi where the levels of gene expression were comparable to mock-infected samples. The upregulation of *chIFITM1* was the largest in both M41-CK- and QX-infected trachea (fold change >70 and >40, respectively) (Figure 3D), followed by *chIFITM3* (fold change >18 and >7, respectively) (Figure 2F), *chIFITM5* (fold change >14 and >8, respectively) (Figure 2G) and *chIFITM2* (fold change >12 and >6, respectively) (Figure 2E). Overall, the mRNA expression of each *chIFITM* was higher in M41-CK-infected tissues when compared to QX, however, this difference was not statistically significant. We observed similar levels and patterns of *IFN* and *chIFITM* expression in M41-CK-infected trachea from both studies (Figure 1 and Figure 2).

In the kidneys of chickens in the same group (kidney samples taken at 2 and 4 dpi only), we only observe low levels of replicating M41-CK at both 2 and 4 dpi (log_10_ M41-CK copies 0.1 × 10 and 0.15 × 10, respectively) (Figure 3A). For QX the viral load was low at 2 dpi (0.8 × 10) but had increased slightly by 4 dpi (1.68 × 10^2^); however, levels were still not significantly different to those of mock-infected samples. We believe that these time points sampled were too early to detect large levels of replicating virus in the kidney. We observe a strikingly different pattern of *IFN* and *chIFITM* mRNA expression in the kidney when compared to the trachea. The relative mRNA expression of *IFN-α* (Figure 3B) and *IFN-β* (Figure 3C) is higher in the kidney when compared to the trachea, and in the kidney, we observe low levels of *chIFITM* expression when compared to expression levels observed in the trachea (Figure 3D–G and Supplementary Figure S2). At 2 dpi, we observe a higher expression of *chIFITM2* and *3* in kidneys from QX-infected chickens when compared to M41-CK-infected chicken kidney samples. *chIFITM1*, which exhibited the largest upregulation in the trachea, is only slightly elevated in kidney samples from M41-CK- and QX-infected chickens at 2 dpi (Figure 3D). The expression of *chIFITM1* and *chIFITM5* (Figure 3G) is downregulated in kidneys from M41-CK-infected chickens at 2 dpi but expression increases by 4 dpi. There was no downregulation of any *chIFITM* observed in the trachea. *ChIFITM2* and *3* mRNA expression is significantly higher in the kidney from QX-infected chickens at 2 dpi and 4 dpi when compared to mock-infected samples but not significantly different to samples from M41-CK-infected chickens (Figure 3E,F, respectively). Levels of *Mx* mRNA expression was similar between M41-CK- and QX-infected chickens’ kidney samples and significantly upregulated in comparison to mock-infected samples at 2 and 4 dpi (Figure 3H). The upregulation of *IFN-α* and *IFN-β* expression in kidney is high even though the viral titers are low. Therefore, we no longer see any correlation between IFN expression and viral load in the kidney like we had observed in the trachea. However, the relative mRNA expression of the *chIFITMs* in the kidney is low, consistent with the low levels of viral load detected in the kidney.

### 3.3. Transcriptional Profiles of chIFITMs in Ex Vivo TOCs

To comprehensively examine *IFITM* expression in response to IBV an in vitro model would be beneficial. However, both M41-CK and QX have restricted tropism in vitro, with M41-CK only able to propagate in primary CK cells. The S gene of Beau-R confers extended tropism to Beau-R, enabling it to replicate in DF-1 cells, Vero cells and BHK cells [18]. To determine whether Beau-R could be used to investigate *IFITM* expression in vitro, an ex vivo TOC model using time points of 2, 4 and 8 hpi was established. Mirroring the two in vivo experiments, Beau-R and M41-CK were investigated, as well as M41-CK and QX. In the ex vivo TOCs, Beau-R does actively replicate as previously reported [66]. The Beau-R viral load steadily increased from 2 hpi (0.5 log_10_) to 8 hpi (2.03 log_10_) and the M41-CK viral load also increased from 2 hpi (0.7 log_10_) to 8 hpi (4.2 log_10_) (Supplementary Figure S3A). Beau-R and M41-CK viral titers detected at 8 hpi were 603 PFU/mL and 1400 PFU/mL, respectively, (Supplementary Figure S3B). The viral load in Beau-R- and M41-CK-infected TOCs were significantly higher when compared to mock-infected samples at each time point; 2 hpi (*p* = 0.04 and *p* = 0.002, respectively), 4 hpi (*p* = 0.02 and *p* = 0.003, respectively) and 8 hpi (*p* = 0.01 and *p* = 0.009, respectively). Supplementary Figure S3C shows the significant increase in viral load (compared to mock-infected samples) from 2 hpi to 8 hpi for M41-CK and QX strains (2 hpi; *p* = 0.04 and *p* = 0.03, respectively, 4 hpi; *p* = 0.02 and *p* = 0.003, respectively, and 8 hpi; *p* = 0.01 and *p* = 0.009, respectively). A slightly higher increase for the nephropathogenic QX strain was detected when compared to the less pathogenic respiratory M41 strain observed in infected TOCs. The infectivity titer measured as ciliostatic dose (CD_50_) at 8 hpi was 2.3 for M41-CK (*p* = 0.006) and 1.98 for QX (*p* = 0.001) (Supplementary Figure S3D). The log_10_ viral copies of M41-CK at 8 hpi in Study 1 is comparable to the log_10_ viral copies of this virus at 8 hpi in Study 2 (4.2 × 10 and 3.9 × 10, respectively).

We also show there is a higher level of relative mRNA expression of each *chIFITM* in TOCs infected with Beau-R, M41-CK or QX when compared to mock-infected TOCs. However, these levels of expression do not reach levels of statistical significance (Supplementary Figure S4). For each *chIFITM*, the relative mRNA expression is higher in QX-infected TOCs when compared to both Beau-R- and M41-CK-infected TOCs; however, this difference is not significant. Regardless of IBV strain, the most upregulated *chIFITM* response in infected TOCs is at 2 hpi, which decreases by 4 and 8 hpi.

### 3.4. Infectious Bronchitis Virus Infection Induces chIFITM Expression In Vitro

**Figure 4:**
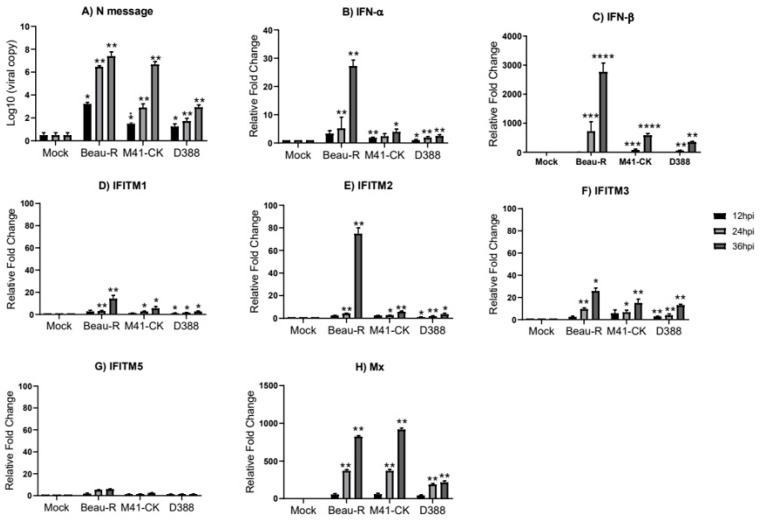
Infection of chick kidney (CK) cells with Beau-R, M41-CK and D388 upregulates the expression of the *chIFITMs and IFNs*. (**A**) Replicating virus detected by qRT-PCR using primers and a probe specific for IBV N message. The expression level and log fold change of (**B**) *IFN-α,* (**C**) *IFN-β*, (**D**–**G**) *chIFITM1*, *2*, *3* and *5* and (**H**) *Mx* were measured using qRT-PCR after infection with Beau-R, M41-CK or D388 (MOI 1) in CK cells normalized to *RPL13* and *RPLPO* housekeeping genes. Error bars show standard deviations of the means (*n* = 3), * indicates *p* < 0.05, ** indicates *p* < 0.01, *** indicates *p* < 0.001, **** indicates *p* < 0.0001. ANOVA data were corrected for multiple comparisons using the Bonferroni adjustment method (GraphPad).

To determine whether IBV infection induces *chIFITM* expression in vitro, CK cells were infected over a 36 h period with the same Beau-R and M41-CK IBV isolates used in the in vivo study. However, the QX strain used for the in vivo experiments cannot be propagated in vitro, therefore the nephropathogenic QX-like strain, D388 [26] was used. For each IBV strain used to infect the CK cells, we observe a steady increase in the viral load from 12 to 36 hpi (Figure 4A). Expression of *IFN-α*, *IFN-β*, and the chicken *IFITMs* (*chIFITM1*, *2* and *3* but not *5*) (Figure 4B–G) are significantly upregulated in response to IBV infection in CK cells. The expression of each *chIFITM* as well as *IFN-α* and *IFN-β* in CK cells infected with the two pathogenic IBV strains, M41-CK and D388, although only moderately upregulated, is significantly higher than what we observe in mock-infected cells. This upregulated expression appears to gradually increase from 12 to 36 hpi. However, the expression of the *IFNs* and *chIFITMs* are upregulated at 36 hpi in CK cells infected with the apathogenic Beau-R strain. Because this increased expression from 24 hpi to 36 hpi is so large, it appears that the upregulation of *IFNs* and *IFITMs* in Beau-R-infected cells is delayed. In CK cells, the expression of the immune genes are maximal at 36 hpi, consistent with the viral load, which also peaks at 36 hpi.

We also employed a chicken DF-1 cell line as a model system to further investigate the interaction between host innate immune system and IBV and the role of IFITMs in defense against viral infection. These further experiments were only performed using Beau-R because the cell tropism of both M41-CK and QX is constrained, limiting their infection to primary CK cultures, which are not suitable for transfection experiments.

**Figure 5:**
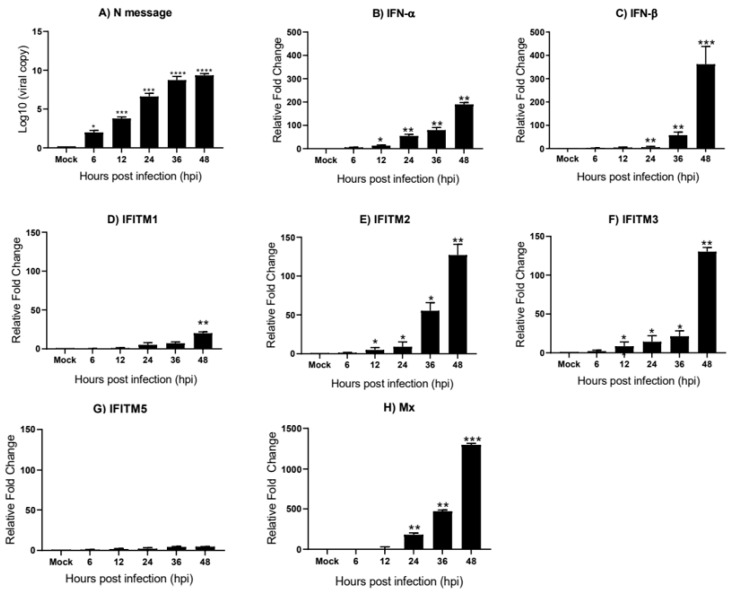
*chIFITM1*, *2*, *3* and *IFNs* are significantly induced in DF-1 cells after Beau-R infection DF-1 cells were infected with MOI of 1 of Beau-R and harvested at 6, 12, 24, 36 and 48 hpi, respectively. (**A**) Replicating virus detected by qRT-PCR using primers and a probe specific for the N message of Beau-R. qRT-PCR was performed to determine the relative mRNA expression of (**B**) *IFN-α*, (**C**) *IFN-β*, (**D**–**G**) *chIFITM1*, *2*, *3*, and *5* and (**H**) *Mx* compared to mock-infected cells. Relative fold change was calculated using the ∆∆CT equation, relative to mock and normalized to two reference genes, *RPL13* and *RPLPO*. Error bars show standard deviations of the means (n = 3). * indicates *p* < 0.05; ** indicates *p* < 0.01; *** indicates *p* < 0.001; **** indicates *p* < 0.0001. ANOVA data were corrected for multiple comparisons using the Bonferroni adjustment method (GraphPad).

In DF-1 cells, we observe a steady increase in the viral load from 6 to 48 hpi (Figure 5A). The relative mRNA expression of *IFN-α* and *IFN-β* were greatly upregulated by Beau-R infection, displaying a substantial increase from 6 to 48 hpi (Figure 5B,C respectively). In comparison to *IFN-α*, which is significantly upregulated by 12 hpi, *IFN-β* appears to have a delayed response with mRNA levels remaining low until 24 hpi, after which it is strongly upregulated reaching 350-fold at 48 hpi. *ChIFITM2* and *3* were highly upregulated by 120-fold and 130-fold at 48 hpi (Figure 5E,F, respectively). The expression of *chIFITM1* (Figure 5D) was upregulated in response to Beau-R infection but only reached levels of significance at 48 hpi. Although *chIFITM5* (Figure 5G) expression was upregulated in response to Beau-R infection, this upregulation did not reach levels of significance. Immune gene expression peaks at 48 hpi, which is consistent with the viral load, which also peaks at 48 hpi.

## 4. Discussion

In this study, we found that key IFNs and immune-related *IFITMs* were significantly upregulated in response to IBV infection in vivo and in vitro. It is well known that IFITMs serve as critical effector molecules in the host innate immune system and are the first line of defense against invading viruses [43,52,72]. The IFN-induced expression of these genes indicates the initiation of the innate host response. These proteins play an important role in the control of infection by influenza [72,73]; respiratory syncytial virus (RSV) [74] as well as mammalian coronaviruses such as the highly pathogenic MERS-CoV [75] and SARS-CoV [38]. Wrensch and colleagues observed that the IFITM-mediated inhibition of cellular entry of SARS-CoV and MERS-CoV was less efficient than blocking the cellular entry of the globally circulating human coronaviruses 229E and NL63, highlighting the differential IFITM-sensitivity of coronaviruses [75]. It would be interesting to determine whether SARS-CoV-2 cell entry and replication is affected in any way by *IFITM* expression. In contrast, Zhao et al., have shown that human IFITM2 and IFITM3 are essential host factors that facilitate the cellular entry of human coronavirus OC43 [76]. The restrictive role of these proteins in the avian coronavirus, IBV, is less well defined. This study is the first to focus primarily on the response of chicken *IFITMs* during early phase infectious bronchitis viral infection, examining the relative mRNA expression profile of *chIFITMs* following IBV infection in vivo and in vitro.

We did not observe any significant upregulation of the *chIFITMs* (or *IFNs*) in the trachea taken from birds infected with the nonpathogenic Beau-R strain (Figure 1). There was also no detection of replicating virus in this tissue suggesting that Beau-R fails to consistently produce a productive infection in vivo. This finding is in line with data from other studies [20,58,68]. The pathogenic M41-CK and nephropathogenic QX strain both actively replicate in the trachea of infected chickens, as is evident by the high viral load detected in this tissue (Figure 2). Infection of chickens with M41-CK and QX strains elicits a significant upregulation of *chIFITM1*, *2*, *3* and *5* in the trachea at 2 and 4 dpi. The expression levels of these *chIFITMs*, in both M41-CK and QX infection, were significantly higher than mock as early as 2 dpi, peaking at 4 dpi, after which they decreased, highlighting a possible role in early response against viral infection. The decrease in immune gene response over time could also be decreasing with the viral load as birds recover from the infection. Although each of the *chIFITMs* had a higher level of expression in the trachea infected with the less virulent M41-CK strain, the difference in gene expression levels between M41-CK and the more virulent QX, did not reach statistical significance. This may suggest that the magnitude of *chIFITM* response is not directly associated with virus pathogenicity, as infection with the more virulent QX strain elicits a smaller *chIFITM* response in the trachea (Figure 2). This observation may be explained by the specific tissue tropism of the different virus strains; M41-CK primarily targets the upper respiratory tract while the nephropathogenic QX exhibits a kidney tropism, therefore potentially eliciting a larger *chIFITM* response in this tissue (Figure 3). We observe a higher level of *chIFITM* expression in QX-infected kidney at 2 dpi in comparison to kidneys from M41-CK-infected birds. Therefore, *chIFITM* response may vary due to specific tissue tropism of the virus rather than differences in the pathogenicity of viral strains. These results could also highlight a tissue-specific response of *chIFITMs*, suggesting that tissue tropism may be a factor that influences *chIFITM* expression. In addition, we cannot rule out potential downregulation of the IFN response by the pathogenic strains of IBV.

We did not detect any M41-CK viral RNA in the kidney tissues sampled at 2 and/or 4 dpi (Figure 3A). This finding is not surprising, as published data suggests that M41 strains may not have the ability to disseminate through the mononuclear cells and plasma at that stage of infection [71], hence this strain is not classified as nephropathogenic. However, some studies have shown that M41 does reach the kidney at low levels, but only after 7 dpi [77,78]. This discrepancy between studies might be a result of variances in the experimental conditions and design, breed or age of the birds and/or the use of different isolates of M41. It is not yet known if the M41-CK isolates of IBV have the ability to infect the kidney in vivo, however, according to our data, we suggest that the absence of any upregulated *chIFITM* response in M41-CK-infected kidney tissue may be due to the fact that there is no virus detected in this tissue at the time points sampled. However, the possibility of the presence of M41-CK viral RNA in the kidney at later time points cannot be completely excluded as they were not sampled beyond 4 dpi in this study. More surprisingly, we observe low levels of the nephropathogenic QX viral RNA in the infected kidney at 4 dpi. These levels are not significantly different to control samples. We believe that the time points sampled may have been too early to detect any productive replication of the virus in the kidneys. In the literature, replicating virus is only detected in the kidneys at or later than 5 dpi [79], 7 dpi [7], 9 dpi [23] and 12 dpi [71]. It is also possible that infection with QX leads to a systemic interferon response whereby tissues with a low viral load, like the kidney, are primed for infection by the initiation of interferon signaling. Although low, the levels of QX viral load detected at 4 dpi is sufficient to elicit a significant upregulation of *chIFITM2* and *3.* The nephropathogenic QX strain and respiratory M41-CK strain have different tissue tropisms; however, both strains successfully replicate in the trachea. Therefore, in vivo, we do not see any correlation between virus pathogenicity with levels of *chIFITM* or *IFN* response, but we do observe a correlation with increasing viral load and immune gene expression.

Tracheal organ cultures (TOCs) have previously been used for examining viruses, including IBV within an in vitro environment [80,81]. Such studies have allowed for the comparison of strain virulence, analysis of infection mechanisms and virus neutralization. In the ex vivo TOC infection study (Supplementary Figures S3 and S4), we observed that all three strains of IBV tested actively replicated in the TOCs, whereas Beau-R did not replicate in the trachea in vivo. Lower infectious virus production was observed for M41-CK-infected TOCs compared to QX-infected TOCs; however, this difference did not reach levels of significance. Transcriptional regulation of *chIFITMs* in TOCs infected with M41-CK and QX strains was analyzed at 8 hpi. No significant upregulation of the *IFITMs* was detected, although *chIFITM1* and *5* mRNA expression were markedly higher in M41-CK-infected TOCs. *chIFITM* expression is highest at 2 hpi followed by a steady decrease over time, suggesting the *chIFITM* response in the TOCs is not sustained as the viral load continues to increase over time. Therefore, in vivo, ex vivo as well as in vitro, we do not see any correlation between increasing viral load and/or the virus pathogenicity with levels of *chIFITM* response. This may suggest that *chIFITM* response may be due to viral entry.

One of the notable impediments in avian viral research is the relatively limited tissue tropism which affects the ability of avian viruses to infect cells in vitro. Therefore, it is not always possible to perform all in vitro experiments in the same cells because of the limited tropism of specific viral strains. This is a recognized issue with coronavirus IBV. In this study we were not able to assess the effect of QX infection on *chIFITM* expression in CK cells as QX does not infect these cells, however, the D388 strain does. D388 is a good substitute for in vitro work with QX because it produces very comparable disease in vivo and it is of the same genotype. In the in vitro studies, we observed that infection with Beau-R, M41-CK and D388 elicit significant upregulation of *IFN-α*, *IFN-β* and *chIFITMs*. Interferon and *chIFITM* gene response to M41-CK and D388 infection in CK cells displayed a gradual increase in expression from 12 hpi to 36 hpi, with levels reaching statistical significance at 24 hpi while in Beau-R-infected CK cells, *IFITM* and *IFN* response is relatively low and at similar levels at 12 and 24 hpi but an increased *IFITM* and *IFN* response is observed at 36 hpi. (Figure 4). A delayed response in immune genes has been observed in CK cells and DF-1 cells infected with apathogenic Beau-R in previous studies [82]. The observed variability in the magnitude of *chIFITM* mRNA expression between the different IBV strains suggests that *chIFITM* response to IBV infection may be strain-dependent, and tissue tropism may be an influencing factor. The nephropathogenic D388 strain elicits similar levels of *chIFITM* expression to the pathogenic M41-CK strain, while we see the greatest upregulation of *IFN* and *chIFITMs* in response to infection with the apathogenic Beau-R. This may suggest that virus pathogenicity does not play a significant role in *chIFITM* induction during the course of infection. However, Beau-R is a cell adapted strain that replicates much more efficiently than D388 and M41-CK in cell culture. In the M41-CK and D388 infection, results mirror the main findings from the in vivo study; however, the expression patterns of each *chIFITM* differs between the in vivo and in vitro studies. *chIFITM1* is highly upregulated in trachea while only moderately upregulated in cells, while *chIFITM2* and *3* are highly upregulated in cells and only moderately upregulated in trachea, highlighting the cell- and tissue-specific expression of this family of immune genes.

There appears to be a correlation between *chIFITM* expression and the presence of replicating virus in the tissues and cell types analyzed in this study. High viral loads are accompanied by high levels of *chIFITM* expression in the trachea, while low, almost undetectable levels of replicating virus in the kidney is accompanied by low levels of *chIFITM* mRNA. We do not observe the same kind of correlation for the IFNs, which demonstrate high levels of mRNA expression in the kidney when there is a negligible detectable replicating virus. Although this data cannot be supported by statistical analysis as the sample size was too small, it may suggest that the level of *chIFITM* expression may depend on the level of replicating virus.

In this study, we have shown that the level of *chIFITM* expression does not correlate with virus pathogenicity, but the varied expression of *chIFITMs* between the pathogenic and apathogenic strains in vitro may be due to the differences in the growth kinetics of the virus. Such variation may influence the level and pattern of *chIFITM* expression during the course of a specific strain of IBV infection. In addition, individual strains may encode specific mechanisms to evade the host immune system by directly or indirectly regulating the expression of host immune genes. The coronavirus accessory proteins in particular have been identified to have potential roles in mediating IFN responses as well as in host cell translation shut off [83,84,85]. All four accessory proteins have been implicated as pathogenicity factors [85]. Our data suggest *chIFITMs* expression peaks early in infection, regardless of the IBV strain. It is possible that *chIFITMs* play a role in the entry of IBV, which is comparable with what has been observed with other coronaviruses [69,71], and that IBV might be differentially restricted by IFITM proteins in a cell- and tissue-specific manner. In addition, it has been shown that basal levels of expression of immune genes will impact viral replication [84], and the basal level of expression of *chIFITMs* is known to vary among different cell and tissue types.

Therefore, we have revealed that IFITM-induced viral restriction appears to be cell-type- and tissue-dependent, in that the efficiency of entry inhibition by IFITM proteins may depend on the expression level in that specific cell and/or tissue.

## 5. Conclusions

We have shown that *chIFITMs* are significantly upregulated in response to IBV infection in vivo and in vitro. Taken together, IBV infection induces an effective host antiviral immune response involving the immune-related IFITM proteins, which may be useful for developing new antiviral drugs in the future.

## Supplementary Materials

The following are available online at https://www.mdpi.com/2073-4425/11/8/918/s1, Figure S1: Schematic overview of the study design, Figure S2: Relative expression of *IFNs* and *chIFITMs* in trachea and kidney samples from chickens infected with M41-CK and the nephropathogenic QX strain, Figure S3: Characterization of Beau-R, M41-CK and QX in tracheal organ cultures (TOCs), Figure S4: Relative expression of *chIFITMs* in IBV infected TOCs.
