# Supplementary material for: The Characterization of chIFITMs in Avian Coronavirus Infection In Vivo, Ex Vivo and In Vitro
Source: Genes (Basel). 2020 Aug 10;11(8):918. doi: 10.3390/genes11080918 (PMC7464837; doi:10.3390/genes11080918)
Supplement: Supplementary file 1 [file genes-11-00918-s001.zip › genes-888973-supplementary.docx]

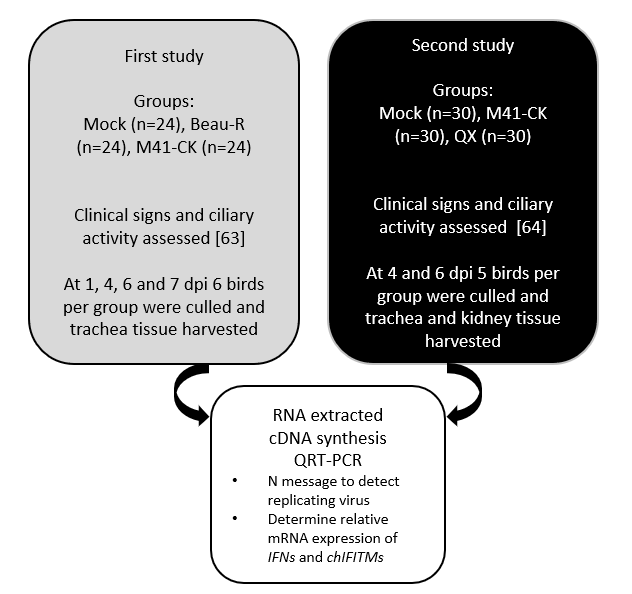


**Supplementary Figure 1.** Schematic overview of the study design.


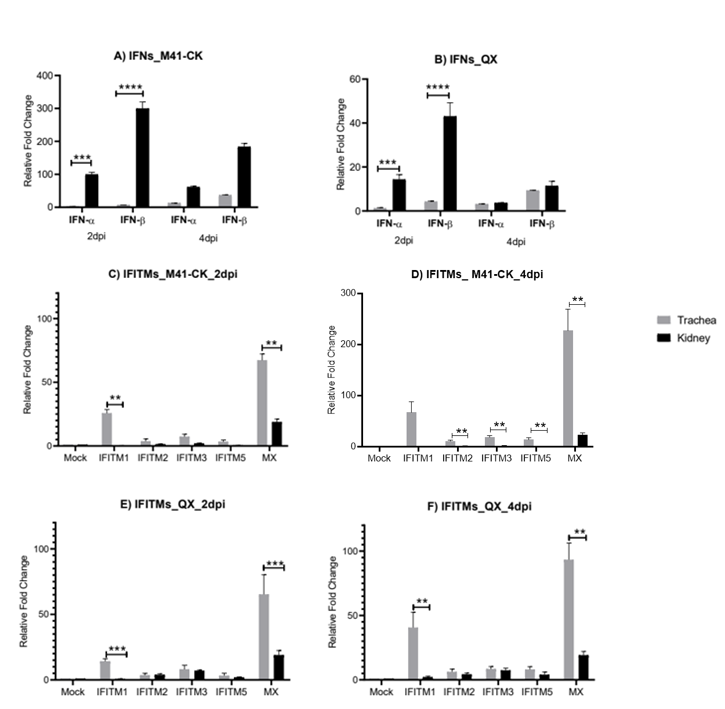


**Supplementary Figure 2.** Relative expression of *IFNs* and *chIFITMs* in trachea and kidney samples from chickens infected with M41-CK and the nephropathogenic QX strain. Relative expression of (A) *IFN-α*, (B) *IFN-β* and (C-F) *chIFITM1*, *2*, *3* and *5* measured by qRT-PCR in tracheal and kidney samples collected at 2 and 4 dpi from chickens experimentally challenged with M41-CK and QX IBV, or mock infected. Relative fold change was calculated using the ∆∆CT equation, relative to mock and normalized to two reference genes, *RPLPO* and *RPL13*. All graph values are the mean of three biological replicates with error bars as standard deviation; target gene expression between the different tissues was compared * indicates *p* < 0.05, ** indicates *p* < 0.01.


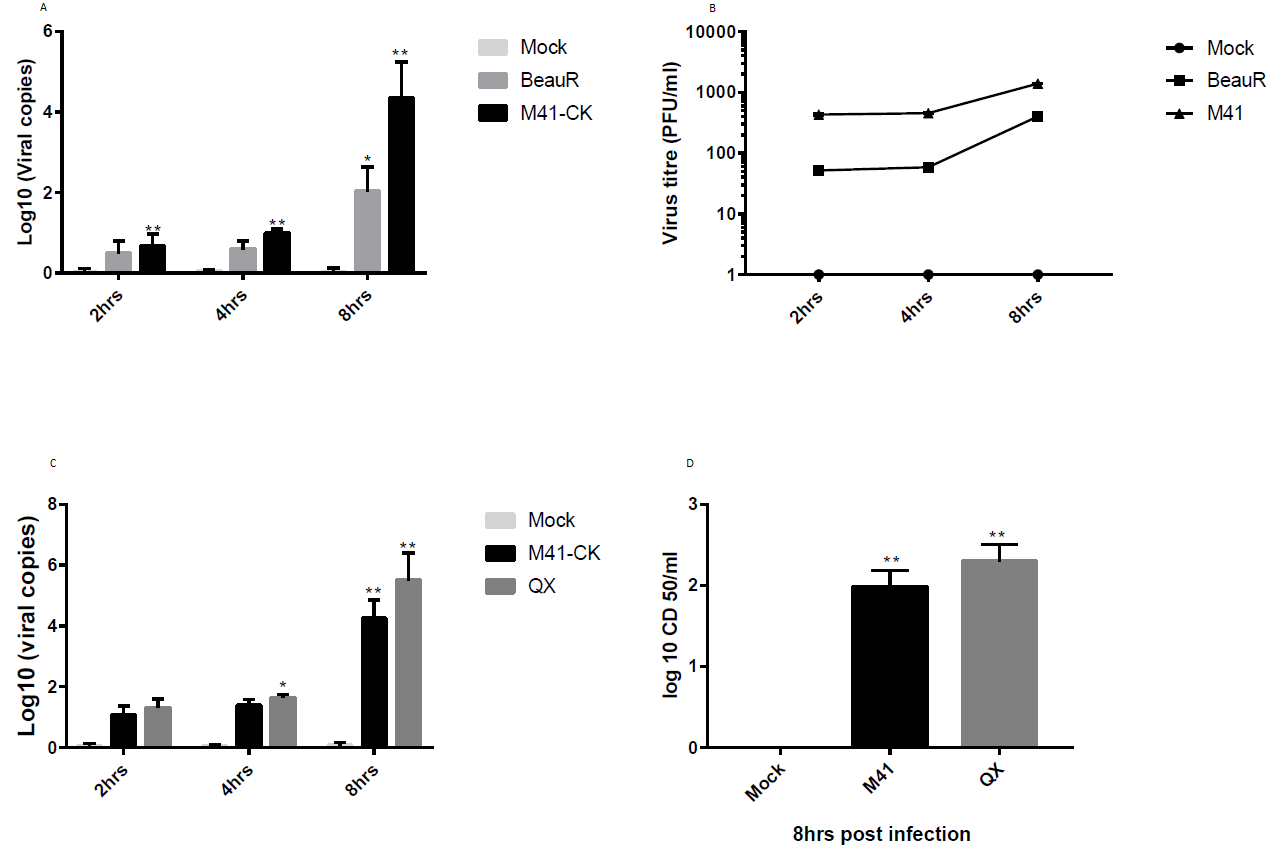


**Supplementary Figure 3**. Characterization of Beau-R, M41-CK and QX in tracheal organ cultures (TOCs). (A) Level of replicating Beau-R and M41-CK in TOCs. (B) TOCs were infected with Beau-R and M41-CK and the supernatants harvested at 2, 4 and 8 h intervals. Release of progeny virus was determined by plaque assay. Data represent the means and standard deviations of the results of three independent experiments. (C) Levels of replicating M41-CK and QX in TOCs, (D) TOCs were inoculated with M41-CK and QX and the supernatants harvested at 8 h. Virus titers were determined in terms of CD50. Data represent the means and standard deviations of the results of three independent experiments. GraphPad Prism was used to test significance between the mock group and infected groups. * indicates *p* < 0.05, ** indicates *p* < 0.01, *** indicates *p* < 0.001, **** indicates *p* < 0.0001. (ANOVA).


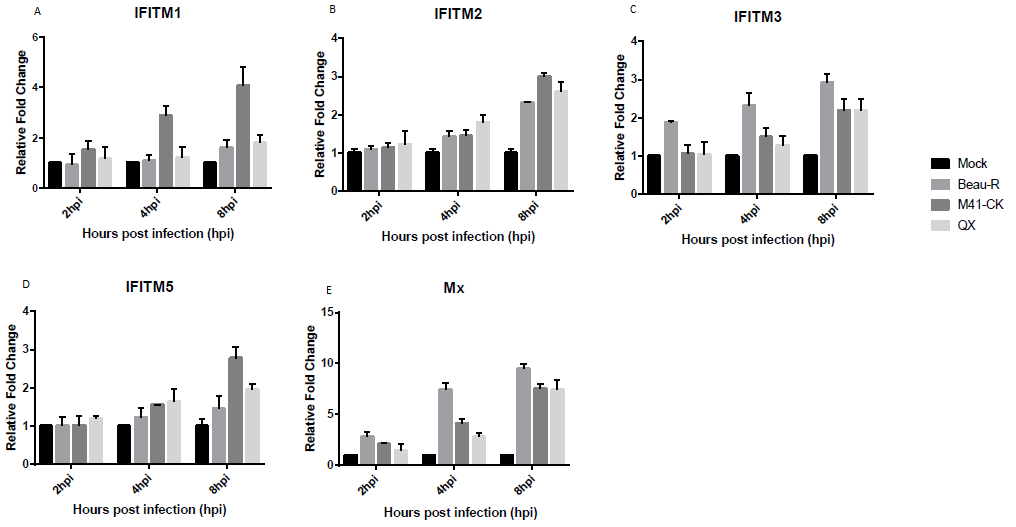


**Supplementary Figure 4.** Relative expression of *chIFITMs* in IBV-infected TOCs. Relative expression of *chIFITM1, 2, 3* and *5* (and positive control, Mx) measured by qRT-PCR in TOCs infected with Beau-R, M41-CK and QX, or mock infected. Relative fold change was calculated using the ∆∆CT equation, relative to mock and normalized to two reference genes, *RPLPO* and *RPL13*.
